# *Plasmodium vivax* metacaspase 1 (*Pv*MCA1) catalytic domain is conserved in field isolates from Brazilian Amazon

**DOI:** 10.1590/0074-02760200584

**Published:** 2021-05-31

**Authors:** Hugo Amorim dos Santos de Souza, Victor Fernandes Escafa, Carolina Moreira Blanco, Bárbara de Oliveira Baptista, Jenifer Peixoto de Barros, Evelyn Ketty Pratt Riccio, Aline Beatriz Mello Rodrigues, Gisely Cardoso de Melo, Marcus Vinícius Guimarães de Lacerda, Rodrigo Medeiros de Souza, Josué da Costa Lima-Junior, Ana Carolina Ramos Guimarães, Fabio Faria da Mota, João Hermínio Martins da Silva, Cláudio Tadeu Daniel-Ribeiro, Lilian Rose Pratt-Riccio, Paulo Renato Rivas Totino

**Affiliations:** 1 Fundação Oswaldo Cruz-Fiocruz, Instituto Oswaldo Cruz, Laboratório de Pesquisa em Malária, Rio de Janeiro, RJ, Brasil; 2 Fundação Oswaldo Cruz-Fiocruz, Instituto Oswaldo Cruz, Laboratório de Genômica Funcional e Bioinformática, Rio de Janeiro, RJ, Brasil; 3 Universidade do Estado do Amazonas, Manaus, AM, Brasil; 4 Fundação de Medicina Tropical Heitor Vieira Dourado, Instituto de Pesquisa Clínica Carlos Borborema, Manaus, AM, Brasil; 5 Fundação Oswaldo Cruz-Fiocruz, Instituto Leônidas and Maria Deane, Manaus, AM, Brasil; 6 Universidade Federal do Acre, Centro de Pesquisa em Doenças Infecciosas, Centro Multidisciplinar, Rio Branco, AC, Brasil; 7 Fundação Oswaldo Cruz-Fiocruz, Instituto Oswaldo Cruz, Laboratório de Imunoparasitologia, Rio de Janeiro, RJ, Brasil; 8 Fundação Oswaldo Cruz-Fiocruz, Instituto Oswaldo Cruz, Laboratório de Biologia Computacional e Sistemas, Rio de Janeiro, RJ, Brasil; 9 Fundação Oswaldo Cruz-Fiocruz, Eusébio, CE, Brasil

**Keywords:** P. vivax, metacaspase, genetic diversity

## Abstract

In the present study, we investigated the genetic diversity of *Plasmodium vivax* metacaspase 1 (*Pv*MCA1) catalytic domain in two municipalities of the main malaria hotspot in Brazil, i.e., the Juruá Valley, and observed complete sequence identity among all *P. vivax* field isolates and the Sal-1 reference strain. Analysis of *Pv*MCA1 catalytic domain in different *P. vivax* genomic sequences publicly available also revealed a high degree of conservation worldwide, with very few amino acid substitutions that were not related to putative histidine and cysteine catalytic residues, whose involvement with the active site of protease was herein predicted by molecular modeling. The genetic conservation presented by *Pv*MCA1 may contribute to its eligibility as a druggable target candidate in vivax malaria.

The emergence and spread of drug-resistant parasites constitute major obstacles to malaria control in the world,^1^ where *Plasmodium falciparum* and *P. vivax* are the most important species causing malaria.^2^ Throughout the history of antimalarial therapy, *P. falciparum* has been notorious for its capacity to develop resistance to diverse antimalarial drugs, including chloroquine, sulfadoxine/pyrimethamine and, more recently, artemisinin in Southeast Asia.^1^ ^,^ ^3^ Nevertheless, emergence of drug-resistant *P. vivax* has also been documented since the late 1980s in Papua New Guinea and,^4^ ^,^ ^5^ currently, therapeutic failure of chloroquine, which combined with primaquine comprise the first-line treatment for vivax malaria, is reported in many vivax-endemic countries,^6^ ^,^ ^7^ while the magnitude of primaquine tolerance remains largely unknown.^8^ These facts highlight the urgent need for the development of novel compounds targeting plasmodial molecular pathways with mechanisms of action different from the classical antimalarial drugs.

In this scenario, proteases playing significant roles in parasite survival have been for long time considered molecular targets for antimalarial drug development and,^9^ ^,^ ^10^ more recently, members of metacaspase family, whose activity was already suggested to be implicated in both growth and cell death of *Plasmodium* and other protozoan parasites, have emerged as novel candidates.^11^ ^,^ ^12^ Metacaspases are cysteine proteases belonging to the C14 family that are found in genome of protists, fungi, algae and plants. They present structural similarity to metazoan caspases, both having a conserved His-Cys catalytic dyad in the large (p20) domain, but different substrate specificity.^13^ In *Plasmodium* genomes, three metacaspases (MCA1-3) occur^14^ and a study performed with field isolates from Mauritania, Sudan and Oman showed that *P. vivax* metacaspase 1 (*Pv*MCA1) can present single nucleotide polymorphisms in the putative His-Cys catalytic residues,^15^ which in some extent can be a factor limiting the eligibility of *Pv*MCA1 as a novel drug target for vivax malaria. In the present study, therefore, we investigated the genetic diversity of the *Pv*MCA1 catalytic domain in a *P. vivax* population from the main malaria hotspot in Brazil - a country where this plasmodial species is highly prevalent (~90%).^2^ ^,^ ^16^

*Study area and sample collection* - *P. vivax* parasite isolates were collected from two municipalities [Cruzeiro do Sul (CZS; 7º37′51″S, 72º40′12″W) and Mâncio Lima (ML; 07º36′50″S, 72º53′4″W)] situated in the Juruá Valley, Northwest of the Acre State, Brazilian Amazon, from June to August 2016 and 2018. Covering together a surface area of 12,597 km^2^ (CZS: 7,925 km^2^; ML: 4,672 km^2^), these municipalities are among the endemic areas with the highest annual parasite incidence (API) (positive blood slides per 1,000 inhabitants) in Brazil, presenting an epidemiological profile with sustained high transmission over the last decade. For reference, the Brazilian Ministry of Health considers high risk areas those with API ≥ 50, and Mâncio Lima and Cruzeiro do Sul registered APIs of 436.4 and 231.9 in 2016 and 422.8 and 147.5 in 2018, respectively. In these areas, eighty-three *P. vivax* mono-infected individuals living in different localities and presenting uncomplicated malaria were diagnosed through microscopic examination of Giemsa-stained thick blood smears and, then, a single blood sample was collected in EDTA tubes from each individual before initiation of treatment. Samples were centrifuged at 350 g for 10 min to remove plasma and cell pellet was preserved at -20ºC in glycerolyte solution (1:2). Written informed consent was obtained from all donors and the study was reviewed and approved by the Oswaldo Cruz Foundation Ethical Committee and the National Ethical Committee of Brazil (CEP-FIOCRUZ CAAE 46084015.1.0000.5248). All *P. vivax* infections were later confirmed by polymerase chain reaction (PCR) assay, as previously described.^17^

*DNA extraction and PCR amplification* - parasite DNA was extracted from cryopreserved blood samples by QIAamp DNA blood midi kit (QIAgen), following the manufacturer’s instructions, and stored at -20ºC until use. Gene segment coding for the catalytic domain of *Pv*MCA1 was amplified by standard PCR method using a pair of specific primers (forward, 5′-CATGGAAACAAAAAAAAGG-3′; reverse, 5′-CGAAAACTCCATATCTTTGC-3′), as previously described.^15^ PCR was performed in a Veriti 96-well Thermal Cycler (Applied Biosystems) with a total volume of 25 μL reaction mixture containing 3 μL genomic DNA, 10 pmol/μL each primer, 2.5 unit AmpliTaq^TM^ Gold DNA polymerase, 3 mM MgCl_2_ and 2 μL of 10X PCR buffer. The following conditions were used: 35 cycles at 95ºC for 30 s, 56ºC for 30 s and 72ºC for 2 min. Amplified products were size-fractionated by electrophoresis within 2% agarose gel (Sigma) containing 0.5 μg/mL of ethidium bromide and, then, were visualised by ultraviolet illumination.

*Sequencing and polymorphism analysis* - PCR products were purified by Wizard^®^ SV Gel and PCR Clean-Up System (Promega) following the manufacturer’s instructions and sequenced in both directions using above-mentioned primers. The sequencing reaction was performed in duplicate according BigDye™ Terminator v3.1 Cycle Sequencing Kit Applied Biosystems™ using 75-100 ng of the purified PCR products and, then, the obtained products were read on a 3730 xl DNA Analyser (Applied Biosystems). Forward and reverse sequences were compared and checked for quality by using SeqMan v.7.0.0 of the DNASTAR software package (Lasergen, Madison, WI, USA), with default parameters, following manual inspection of chromatograms to eliminate ambiguous bases. A minimum quality score of 20 (base call accuracy ≥ 99%) was considered. Alignment of edited sequences was performed in MEGA 7 using Clustal X2 algorithm to identify polymorphisms relative to *Pv*MCA1 sequence from El Salvador reference strain (Sal-1; PlasmoDB: PVX_114725). Additionally, for worldwide analysis, 112 nucleotide sequences for the complete catalytic domain of *Pv*MCA1, as predicted using Pfam database (https://pfam.xfam.org/), were recovered from PlasmoDB and GenBank databases by BLAST and aligned to search for genetic variability. Metadata obtained were used to identify geographical distribution of the isolates recovered from these databases.

*Molecular modeling* - The sequence of G0ZIA8 (Sal-1; UniProtKB) was submitted to Blast, in the search of homologous proteins. The template (4AFR, *Trypanosoma brucei* metacaspase) found in PDB share 38.4% identity with G0ZIA8. After alignment with blast, the coverage is 32%. The e-value was 7e-35. The structural model of G0ZIA8 was constructed with MODELLER (https://salilab.org/modeller/). Fifty models were constructed and the best one was selected according to the DOPE score. The electrostatic surface potential was calculated by APBS software. The structural analysis was performed with Pymol.

To investigate the genetic diversity of *Pv*MCA1, eighty-three wild isolates of *P. vivax* from Juruá Valley in the Brazilian Amazon were submitted to DNA sequencing for gene segment coding the consensus His372-Cys428 catalytic dyad (13). In contrast to work by Sow et al.,^15^ in which the majority of isolates (24/28, 85.7%) presented amino acid substitution in both catalytic dyad and an upstream cysteine residue (Cys305) also inserted in the peptidase C14 domain, all the isolates herein studied showed complete nucleotide sequence identity each other as well as compared to *P. vivax* Sal-1 reference, with conservation of all the amino acid residues successfully sequenced (A288 to K446) (Fig. 1), supporting that the proteolytic activity of *Pv*MCA1 may be critical to the parasite.

**Fig. 1:**
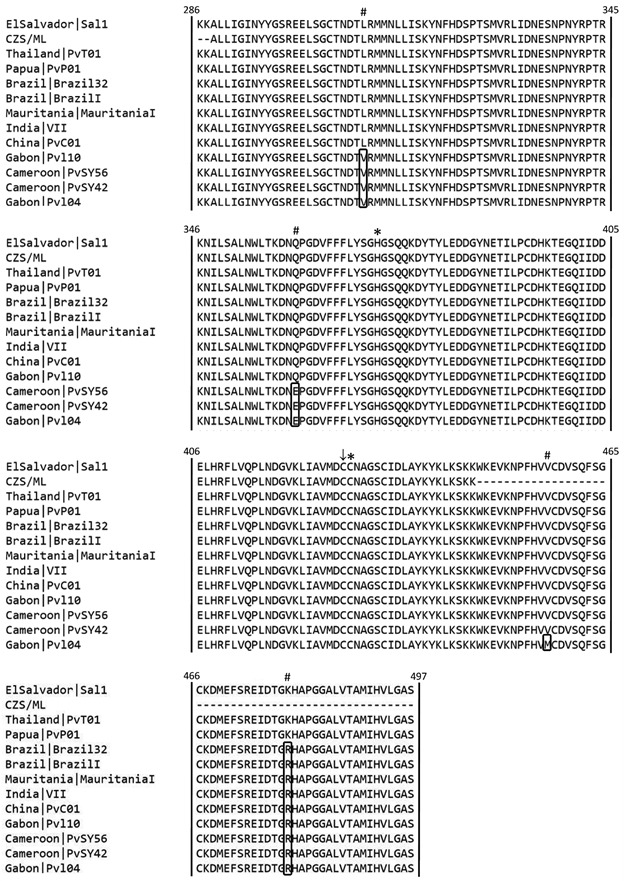
multiple alignment of *Pv*MCA1 peptidase domain from Brazilian Amazon field isolates and *Plasmodium vivax* strains from different endemic countries around the world. Deduced amino acid sequences of Peptidase_C14 domain of *Pv*MCA1 were obtained from 83 *P. vivax* isolates collected in two municipalities of the Juruá Valley (CZS: Cruzeiro do Sul and; Mâncio Lima: ML) and were, then, compared against sequences deduced from 112 *P. vivax* nucleotide genomic sequences available in GenBank and PlasmoDB, using Sal-1 as reference strain. Selected sequences are shown and the complete analysis is available in Supplementary data. The canonical His372-Cys428 catalytic dyad and the substitutions of amino acid residues are indicated by (*) and (#), respectively. (↓) indicates the adjacent cysteine residue (Cys427) with possible involvement in protease activity. CZS/ML represents all field isolates studied, since complete identity was observed. Monkey-adapted strains are represented by Sal1, BrazilI, MauritaniaI and IndiaVII; clinical isolates by Brazil32, PvC01, PvT01 and PvP01 and; wild ape isolates by PvSY42, PvSY56, Pv104 and Pvl10. (-): indicates non-determined amino acid residues.

Since functional disruption of the single metacaspase (MCA1) in *Saccharomyces cerevisiae* by mutagenesis was already shown to induce *in vitro* drug-resistance as a result of refractoriness to apoptosis in the yeasts,^18^ one possibility is that polymorphism of *Pv*MCA1 could be related to chemoresistance phenomena reported in vivax malaria, whose occurrence is reported to be infrequent in Juruá Valley^19^ and that was not characterised in our cross-sectional study. Indeed, metacaspases have been implicated in apoptosis-like cell-death pathways in different microorganisms^20^ and drug-resistant strains of *P. falciparum* seem to be insensitive to choroquine-induced apoptosis^21^ as well as to anti-cell death effect promoted by inhibition of metacaspase activity.^22^ Nevertheless, our preliminary study with recurrent infection in five *P. vivax* patients receiving appropriate antimalarial therapy (chloroquine/primaquine) at a Reference Center for malaria in Manaus city, Amazonas state, does not indicate a relation between *Pv*MCA1 genetic diversity and parasite chemoresistance, as no mutation in the protease catalytic domain was detected in these patients (data not shown). Even so, the conclusion of the survey with a larger number of samples appropriately characterised in terms of parasite drug-sensitivity is needed to better clarify this issue.

In order to know the variability of *Pv*MCA1 worldwide, we extended our analysis to *P. vivax* isolates from different geographical regions whose genomic sequences are publicly available and, additionally, evaluated complete peptidase C14 domain (K286-S497), as predicted using Pfam database of protein families. Nucleotide sequence analysis of more than one hundred isolates from Americas, Africa, Asia and Indonesia, which included clinical and monkey-adapted strains as well as ape-infecting strains from Cameroon and Gabon (Supplementary data), revealed only four non-synonymous polymorphisms at position 310 (Leu to Val), 360 (Gln to Glu), 457 (Val to Met) and 479 (Lys to Arg) (Fig. 1). Three of them (L310V, Q360E, V457M) were restricted to wild ape parasites, with L310V substitution occurring markedly in all eight isolates analysed, while K479R substitution was the unique variation observed in the clinical and monkey-adapted isolates (Fig. 1 and Supplementary data). It is noteworthy that substitutions were not related to cysteine and histidine residues; even in the ape-infecting *P. vivax* strains, whose remarkable genetic diversity comparing to human strains suggests distinct demographic histories.^23^ These data indicate, therefore, that the peptidase domain of *Pv*MCA1 presents evolutionary conservation of amino acid residues that are putatively involved in metacaspase activity, as described across diverse taxa.^24^ ^,^ ^25^

Similar results showing few polymorphic nucleotides with high conservation of essential amino acid residues required to catalytic site formation have previously been reported for other *P. vivax* proteases, such as vivapain-1, -2 and -3, plasmepsin-4 and *Pv*SERA-4.^26^ ^,^ ^27^ ^,^ ^28^ Curiously, the polymorphisms reported by Sow et al.^15^ in *Pv*MCA1 peptidase domain were restricted to histidine and cysteine residues, including the putative H372-C428 catalytic dyad, which could impact protease activity. Abrogation of proteolytic activity can indeed be achieved by site-specific mutagenesis of histidine and cysteine residues forming consensus catalytic dyad in metacaspases of *T. brucei* (*Tb*MCA2), *Leishmania major* (*Lm*jMCA) and *Candida albicans* (*Ca*Mca1).^29^ ^,^ ^30^ ^,^ ^31^ But in other metacaspases this dyad seems not to be essential for catalytic activity, such as in *Ld*MCA1 of *L. donovani*,^32^ and a secondary catalytic cysteine has been evidenced in *T. congolense* (*Tco*MCA5) as well as in the plant species *Arabidopsis thaliana* (*At*MC9) and *Triticum aestivum* (*Tae*MCAII).^33^ ^,^ ^34^ ^,^ ^35^

This raises the possibility of alternative amino acid residues in *Pv*MCA1 compensating for the mutations previously reported by Sow and colleagues.^15^ For instance, in MCA1 of *P. berghei*, *P. chabaudi* and *P. yoelii* the predicted catalytic cysteine occurs immediately before of the consensus position, where a substitute residue (proline) is present.^14^ The same is true for *T. brucei* MCA4 (*Tb*MCA4), in which the activity of such cysteine was experimentally demonstrated.^36^ Interestingly, *Pv*MCA1 (Fig. 1; Cys427) and other metacaspases, including MCA1 of *P. knowlesi* and *P. gallinaceum*, present a second conserved cysteine adjacently preceding the consensus catalytic cysteine^11^ ^,^ ^14^ and the involvement in proteolytic activity has been shown as well.^29^ ^,^ ^30^ ^,^ ^35^ Notably, substitution of this alternative cysteine is not found in any of the *P. vivax* isolates analysed until now (Cys427), indicating by its high degree of conservation a possible role in *Pv*MCA1 activity.

We then predicted the three-dimensional structure of *Pv*MCA1 catalytic domain by homology modeling to examine if C427 and additional histidine residues could, in some extent, participate in catalytic site formation. As shown in Fig. 2, the area harboring the canonical catalytic dyad (His372-Cys428) presented typical electrostatic potential of metacaspase catalytic pocket (Fig. 2B), which is endowed with negative charges and confers specificity toward substrates containing basic arginine or lysine residues,^13^ ^,^ ^24^ while Cys427 was located in a more positively charged area (Fig. 2B). On the other hand, the proximity of both Cys427 (7.8 Å) and Cys428 (6.1 Å) to His372 residue was shown to be consistent with the active site reported for some cysteine proteases (Fig. 2A), such as legumains, caspases and metacaspases.^34^ ^,^ ^37^ ^,^ ^38^ In the single metacaspase of *S. cerevisiae* (YCA1), for instance, the distance between catalytic residues (Cys-His) was about 9.2 Å, while the nearest histidine (5.8 Å) was not implicated in the proteolytic process,^35^ supporting that Cys427 could participate in the activity of *Pv*MCA1. In this context, an additional histidine residue (His480) was also identified in the vicinity of the canonical catalytic dyad but, unless a conformational change occurs before catalytic site activity, His480 does not seem to constitute a partner residue for Cys427 or Cys 428, since it is >13.0 Å away (Fig. 2A).

**Fig. 2:**
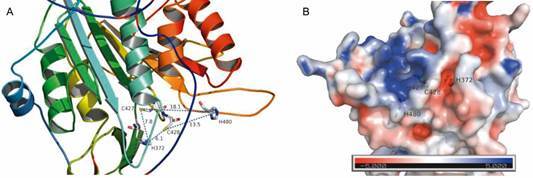
prediction of catalytic dyad of *Pv*MCA1 by molecular modeling. (A) Three-dimensional structure of *Pv*MCA1 catalytic domain was modeled using comparative modeling and the distances (in Å) between the putative amino acid residues participating in catalytic dyad formation were estimated considering the Nε atom of histidine (H) imidazole ring and Sγ atom of cysteine (C), which are crucial for the proteolytic process. (B) Electrostatic surface potential analysis of *Pv*MCA1 catalytic domain: negatively charged regions are shown in red and positively regions in blue.

In summary, although mutations in the putative catalytic dyad of *Pv*MCA1 have previously been reported in field isolates from Mauritania, Sudan and Oman, a highly conserved peptidase domain of *Pv*MCA1 was remarkably observed in Brazilian Amazon as well as between *P. vivax* isolates from different endemic countries around the world, which can contribute to eligibility of this metacaspase as a druggable target candidate in vivax malaria. Nevertheless, further studies aiming biochemical and functional characterisation of *Pv*MCA1, together to identification of amino acid residues that effectively participate in the proteolytic process are still required to elucidate the role of *Pv*MCA1 in *P. vivax* biology.
