# Supplementary material for: Plasmodium vivax metacaspase 1 (PvMCA1) catalytic domain is conserved in field isolates from Brazilian Amazon
Source: Mem Inst Oswaldo Cruz. 2021 May 31;116:e200584. doi: 10.1590/0074-02760200584 (PMC8186469; doi:10.1590/0074-02760200584)
Supplement: Supplementary file 1 [file 1678-8060-mioc-116-e200584-s.pdf]

	286 345
ElSalvador|Sal1	KKALLIGINYYGSREELSGCTNDTLRMMNLLISKYNFHDSPTSMVRLIDNESNPNYRPTR
Colombia|30102100437	KKALLIGINYYGSREELSGCTNDTLRMMNLLISKYNFHDSPTSMVRLIDNESNPNYRPTR
Colombia|30102100490	KKALLIGINYYGSREELSGCTNDTLRMMNLLISKYNFHDSPTSMVRLIDNESNPNYRPTR
Colombia|30102100438-B	KKALLIGINYYGSREELSGCTNDTLRMMNLLISKYNFHDSPTSMVRLIDNESNPNYRPTR
Colombia|30102100438-A	KKALLIGINYYGSREELSGCTNDTLRMMNLLISKYNFHDSPTSMVRLIDNESNPNYRPTR
Colombia|30101099036	KKALLIGINYYGSREELSGCTNDTLRMMNLLISKYNFHDSPTSMVRLIDNESNPNYRPTR
Colombia|30101099040	KKALLIGINYYGSREELSGCTNDTLRMMNLLISKYNFHDSPTSMVRLIDNESNPNYRPTR
Colombia|30102100486	KKALLIGINYYGSREELSGCTNDTLRMMNLLISKYNFHDSPTSMVRLIDNESNPNYRPTR
Colombia|30111110026	KKALLIGINYYGSREELSGCTNDTLRMMNLLISKYNFHDSPTSMVRLIDNESNPNYRPTR
Colombia|30103103280	KKALLIGINYYGSREELSGCTNDTLRMMNLLISKYNFHDSPTSMVRLIDNESNPNYRPTR
Mexico|330-A	KKALLIGINYYGSREELSGCTNDTLRMMNLLISKYNFHDSPTSMVRLIDNESNPNYRPTR
Mexico|938-A	KKALLIGINYYGSREELSGCTNDTLRMMNLLISKYNFHDSPTSMVRLIDNESNPNYRPTR
Mexico|Mexico_161-04	KKALLIGINYYGSREELSGCTNDTLRMMNLLISKYNFHDSPTSMVRLIDNESNPNYRPTR
Mexico|55-03	KKALLIGINYYGSREELSGCTNDTLRMMNLLISKYNFHDSPTSMVRLIDNESNPNYRPTR
Mexico|1086-A	KKALLIGINYYGSREELSGCTNDTLRMMNLLISKYNFHDSPTSMVRLIDNESNPNYRPTR
Mexico|203-04	KKALLIGINYYGSREELSGCTNDTLRMMNLLISKYNFHDSPTSMVRLIDNESNPNYRPTR
Mexico|63-08	KKALLIGINYYGSREELSGCTNDTLRMMNLLISKYNFHDSPTSMVRLIDNESNPNYRPTR
Mexico|21-A	KKALLIGINYYGSREELSGCTNDTLRMMNLLISKYNFHDSPTSMVRLIDNESNPNYRPTR
Mexico|566-A	KKALLIGINYYGSREELSGCTNDTLRMMNLLISKYNFHDSPTSMVRLIDNESNPNYRPTR
Mexico|32-E-03	KKALLIGINYYGSREELSGCTNDTLRMMNLLISKYNFHDSPTSMVRLIDNESNPNYRPTR
Mexico|760-A	KKALLIGINYYGSREELSGCTNDTLRMMNLLISKYNFHDSPTSMVRLIDNESNPNYRPTR
Mexico|118-A	KKALLIGINYYGSREELSGCTNDTLRMMNLLISKYNFHDSPTSMVRLIDNESNPNYRPTR
Mexico|165-A	KKALLIGINYYGSREELSGCTNDTLRMMNLLISKYNFHDSPTSMVRLIDNESNPNYRPTR
Mexico|267-A	KKALLIGINYYGSREELSGCTNDTLRMMNLLISKYNFHDSPTSMVRLIDNESNPNYRPTR
Peru|260	KKALLIGINYYGSREELSGCTNDTLRMMNLLISKYNFHDSPTSMVRLIDNESNPNYRPTR
Peru|858	KKALLIGINYYGSREELSGCTNDTLRMMNLLISKYNFHDSPTSMVRLIDNESNPNYRPTR
Peru|00692	KKALLIGINYYGSREELSGCTNDTLRMMNLLISKYNFHDSPTSMVRLIDNESNPNYRPTR
Peru|07	KKALLIGINYYGSREELSGCTNDTLRMMNLLISKYNFHDSPTSMVRLIDNESNPNYRPTR
Peru|00699	KKALLIGINYYGSREELSGCTNDTLRMMNLLISKYNFHDSPTSMVRLIDNESNPNYRPTR
Peru|DTS0839	KKALLIGINYYGSREELSGCTNDTLRMMNLLISKYNFHDSPTSMVRLIDNESNPNYRPTR
Peru|00622	KKALLIGINYYGSREELSGCTNDTLRMMNLLISKYNFHDSPTSMVRLIDNESNPNYRPTR
Peru|99622	KKALLIGINYYGSREELSGCTNDTLRMMNLLISKYNFHDSPTSMVRLIDNESNPNYRPTR
Peru|ST02	KKALLIGINYYGSREELSGCTNDTLRMMNLLISKYNFHDSPTSMVRLIDNESNPNYRPTR
Peru|08	KKALLIGINYYGSREELSGCTNDTLRMMNLLISKYNFHDSPTSMVRLIDNESNPNYRPTR
Peru|06	KKALLIGINYYGSREELSGCTNDTLRMMNLLISKYNFHDSPTSMVRLIDNESNPNYRPTR
Peru|3136	KKALLIGINYYGSREELSGCTNDTLRMMNLLISKYNFHDSPTSMVRLIDNESNPNYRPTR
Peru|DTS0830	KKALLIGINYYGSREELSGCTNDTLRMMNLLISKYNFHDSPTSMVRLIDNESNPNYRPTR
India|PvHMP3	KKALLIGINYYGSREELSGCTNDTLRMMNLLISKYNFHDSPTSMVRLIDNESNPNYRPTR
Thailand|PvT01	KKALLIGINYYGSREELSGCTNDTLRMMNLLISKYNFHDSPTSMVRLIDNESNPNYRPTR
Papua|PvP01	KKALLIGINYYGSREELSGCTNDTLRMMNLLISKYNFHDSPTSMVRLIDNESNPNYRPTR
Brazil|BrazilI	KKALLIGINYYGSREELSGCTNDTLRMMNLLISKYNFHDSPTSMVRLIDNESNPNYRPTR
Brazil|Brazil29	KKALLIGINYYGSREELSGCTNDTLRMMNLLISKYNFHDSPTSMVRLIDNESNPNYRPTR
Brazil|Brazil32	KKALLIGINYYGSREELSGCTNDTLRMMNLLISKYNFHDSPTSMVRLIDNESNPNYRPTR
Colombia|30102100441-A	KKALLIGINYYGSREELSGCTNDTLRMMNLLISKYNFHDSPTSMVRLIDNESNPNYRPTR
Colombia|30111110015	KKALLIGINYYGSREELSGCTNDTLRMMNLLISKYNFHDSPTSMVRLIDNESNPNYRPTR
Colombia|30102100448	KKALLIGINYYGSREELSGCTNDTLRMMNLLISKYNFHDSPTSMVRLIDNESNPNYRPTR
Colombia|30102100485	KKALLIGINYYGSREELSGCTNDTLRMMNLLISKYNFHDSPTSMVRLIDNESNPNYRPTR
Colombia|30102100446	KKALLIGINYYGSREELSGCTNDTLRMMNLLISKYNFHDSPTSMVRLIDNESNPNYRPTR
Colombia|30111110020	KKALLIGINYYGSREELSGCTNDTLRMMNLLISKYNFHDSPTSMVRLIDNESNPNYRPTR
Colombia|30102100441-B	KKALLIGINYYGSREELSGCTNDTLRMMNLLISKYNFHDSPTSMVRLIDNESNPNYRPTR
Colombia|30102100439	KKALLIGINYYGSREELSGCTNDTLRMMNLLISKYNFHDSPTSMVRLIDNESNPNYRPTR
Colombia|30102100445	KKALLIGINYYGSREELSGCTNDTLRMMNLLISKYNFHDSPTSMVRLIDNESNPNYRPTR
Colombia|30102100440	KKALLIGINYYGSREELSGCTNDTLRMMNLLISKYNFHDSPTSMVRLIDNESNPNYRPTR
Colombia|30102100489	KKALLIGINYYGSREELSGCTNDTLRMMNLLISKYNFHDSPTSMVRLIDNESNPNYRPTR
Colombia|30102100488	KKALLIGINYYGSREELSGCTNDTLRMMNLLISKYNFHDSPTSMVRLIDNESNPNYRPTR
Colombia|30102100504	KKALLIGINYYGSREELSGCTNDTLRMMNLLISKYNFHDSPTSMVRLIDNESNPNYRPTR
Mexico|980-A	KKALLIGINYYGSREELSGCTNDTLRMMNLLISKYNFHDSPTSMVRLIDNESNPNYRPTR
Peru|3133	KKALLIGINYYGSREELSGCTNDTLRMMNLLISKYNFHDSPTSMVRLIDNESNPNYRPTR
Peru|259	KKALLIGINYYGSREELSGCTNDTLRMMNLLISKYNFHDSPTSMVRLIDNESNPNYRPTR
Peru|DTS0721	KKALLIGINYYGSREELSGCTNDTLRMMNLLISKYNFHDSPTSMVRLIDNESNPNYRPTR
Peru|1008	KKALLIGINYYGSREELSGCTNDTLRMMNLLISKYNFHDSPTSMVRLIDNESNPNYRPTR
Peru|3232	KKALLIGINYYGSREELSGCTNDTLRMMNLLISKYNFHDSPTSMVRLIDNESNPNYRPTR
Peru|DTS0791	KKALLIGINYYGSREELSGCTNDTLRMMNLLISKYNFHDSPTSMVRLIDNESNPNYRPTR
Peru|257	KKALLIGINYYGSREELSGCTNDTLRMMNLLISKYNFHDSPTSMVRLIDNESNPNYRPTR
Peru|2025	KKALLIGINYYGSREELSGCTNDTLRMMNLLISKYNFHDSPTSMVRLIDNESNPNYRPTR
Peru|4023	KKALLIGINYYGSREELSGCTNDTLRMMNLLISKYNFHDSPTSMVRLIDNESNPNYRPTR
Madagascar|M15	KKALLIGINYYGSREELSGCTNDTLRMMNLLISKYNFHDSPTSMVRLIDNESNPNYRPTR
Madagascar|M19	KKALLIGINYYGSREELSGCTNDTLRMMNLLISKYNFHDSPTSMVRLIDNESNPNYRPTR
Madagascar|M08	KKALLIGINYYGSREELSGCTNDTLRMMNLLISKYNFHDSPTSMVRLIDNESNPNYRPTR
Mauritania|MauritaniaI	KKALLIGINYYGSREELSGCTNDTLRMMNLLISKYNFHDSPTSMVRLIDNESNPNYRPTR

```
Cambodia|C127              KKALLIGINYYGSREELSGCTNDTLRMMNLLISKYNFHDSPTSMVRLIDNESNPNYRPTR
Cambodia|C08               KKALLIGINYYGSREELSGCTNDTLRMMNLLISKYNFHDSPTSMVRLIDNESNPNYRPTR
Cambodia|C15               KKALLIGINYYGSREELSGCTNDTLRMMNLLISKYNFHDSPTSMVRLIDNESNPNYRPTR
China|PvC01                KKALLIGINYYGSREELSGCTNDTLRMMNLLISKYNFHDSPTSMVRLIDNESNPNYRPTR
China|CMB1                 KKALLIGINYYGSREELSGCTNDTLRMMNLLISKYNFHDSPTSMVRLIDNESNPNYRPTR
China|LZCH-20              KKALLIGINYYGSREELSGCTNDTLRMMNLLISKYNFHDSPTSMVRLIDNESNPNYRPTR
China|NB-16                KKALLIGINYYGSREELSGCTNDTLRMMNLLISKYNFHDSPTSMVRLIDNESNPNYRPTR
China|NB-15                KKALLIGINYYGSREELSGCTNDTLRMMNLLISKYNFHDSPTSMVRLIDNESNPNYRPTR
China|NB-13-1              KKALLIGINYYGSREELSGCTNDTLRMMNLLISKYNFHDSPTSMVRLIDNESNPNYRPTR
China|NB-17                KKALLIGINYYGSREELSGCTNDTLRMMNLLISKYNFHDSPTSMVRLIDNESNPNYRPTR
China|LZCH-4               KKALLIGINYYGSREELSGCTNDTLRMMNLLISKYNFHDSPTSMVRLIDNESNPNYRPTR
China|LZCH-13              KKALLIGINYYGSREELSGCTNDTLRMMNLLISKYNFHDSPTSMVRLIDNESNPNYRPTR
India|VII                  KKALLIGINYYGSREELSGCTNDTLRMMNLLISKYNFHDSPTSMVRLIDNESNPNYRPTR
India|NYC                  KKALLIGINYYGSREELSGCTNDTLRMMNLLISKYNFHDSPTSMVRLIDNESNPNYRPTR
North|NorthKorean          KKALLIGINYYGSREELSGCTNDTLRMMNLLISKYNFHDSPTSMVRLIDNESNPNYRPTR
Thailand|VKBT-106          KKALLIGINYYGSREELSGCTNDTLRMMNLLISKYNFHDSPTSMVRLIDNESNPNYRPTR
Thailand|VKBT-98           KKALLIGINYYGSREELSGCTNDTLRMMNLLISKYNFHDSPTSMVRLIDNESNPNYRPTR
Thailand|VKTS-39           KKALLIGINYYGSREELSGCTNDTLRMMNLLISKYNFHDSPTSMVRLIDNESNPNYRPTR
Thailand|VKTS-36           KKALLIGINYYGSREELSGCTNDTLRMMNLLISKYNFHDSPTSMVRLIDNESNPNYRPTR
Thailand|VKTS-52           KKALLIGINYYGSREELSGCTNDTLRMMNLLISKYNFHDSPTSMVRLIDNESNPNYRPTR
Thailand|VKBT-100          KKALLIGINYYGSREELSGCTNDTLRMMNLLISKYNFHDSPTSMVRLIDNESNPNYRPTR
Thailand|VKTS-45           KKALLIGINYYGSREELSGCTNDTLRMMNLLISKYNFHDSPTSMVRLIDNESNPNYRPTR
Thailand|VKBT-95           KKALLIGINYYGSREELSGCTNDTLRMMNLLISKYNFHDSPTSMVRLIDNESNPNYRPTR
Thailand|VKBT-72           KKALLIGINYYGSREELSGCTNDTLRMMNLLISKYNFHDSPTSMVRLIDNESNPNYRPTR
Thailand|VKBT-94           KKALLIGINYYGSREELSGCTNDTLRMMNLLISKYNFHDSPTSMVRLIDNESNPNYRPTR
Thailand|VKBT-99           KKALLIGINYYGSREELSGCTNDTLRMMNLLISKYNFHDSPTSMVRLIDNESNPNYRPTR
Thailand|VKBT-101          KKALLIGINYYGSREELSGCTNDTLRMMNLLISKYNFHDSPTSMVRLIDNESNPNYRPTR
Thailand|VKBT-71           KKALLIGINYYGSREELSGCTNDTLRMMNLLISKYNFHDSPTSMVRLIDNESNPNYRPTR
Thailand|VKTS-37           KKALLIGINYYGSREELSGCTNDTLRMMNLLISKYNFHDSPTSMVRLIDNESNPNYRPTR
Papua|Chesson              KKALLIGINYYGSREELSGCTNDTLRMMNLLISKYNFHDSPTSMVRLIDNESNPNYRPTR
Papua|PNG58                KKALLIGINYYGSREELSGCTNDTLRMMNLLISKYNFHDSPTSMVRLIDNESNPNYRPTR
Papua|PVRVL1997            KKALLIGINYYGSREELSGCTNDTLRMMNLLISKYNFHDSPTSMVRLIDNESNPNYRPTR
Papua|PNG72                KKALLIGINYYGSREELSGCTNDTLRMMNLLISKYNFHDSPTSMVRLIDNESNPNYRPTR
Papua|XUC014               KKALLIGINYYGSREELSGCTNDTLRMMNLLISKYNFHDSPTSMVRLIDNESNPNYRPTR
Gabon|Pvl10                KKALLIGINYYGSREELSGCTNDTVRMMNLLISKYNFHDSPTSMVRLIDNESNPNYRPTR
Cameroon|PvSY56            KKALLIGINYYGSREELSGCTNDTVRMMNLLISKYNFHDSPTSMVRLIDNESNPNYRPTR
Cameroon|PvSY42            KKALLIGINYYGSREELSGCTNDTVRMMNLLISKYNFHDSPTSMVRLIDNESNPNYRPTR
Cameroon|PvSY43            KKALLIGINYYGSREELSGCTNDTVRMMNLLISKYNFHDSPTSMVRLIDNESNPNYRPTR
Gabon|Pvl06                KKALLIGINYYGSREELSGCTNDTVRMMNLLISKYNFHDSPTSMVRLIDNESNPNYRPTR
Gabon|Pvl11                KKALLIGINYYGSREELSGCTNDTVRMMNLLISKYNFHDSPTSMVRLIDNESNPNYRPTR
Gabon|Pvl03                KKALLIGINYYGSREELSGCTNDTVRMMNLLISKYNFHDSPTSMVRLIDNESNPNYRPTR
Gabon|Pvl04                KKALLIGINYYGSREELSGCTNDTVRMMNLLISKYNFHDSPTSMVRLIDNESNPNYRPTR
                           ************************:***********************************

                          346                                                         405
ElSalvador|Sal1            KNILSALNWLTKDNQPGDVFFFLYSGHGSQQKDYTYLEDDGYNETILPCDHKTEGQIIDD
Colombia|30102100437       KNILSALNWLTKDNQPGDVFFFLYSGHGSQQKDYTYLEDDGYNETILPCDHKTEGQIIDD
Colombia|30102100490       KNILSALNWLTKDNQPGDVFFFLYSGHGSQQKDYTYLEDDGYNETILPCDHKTEGQIIDD
Colombia|30102100438-B     KNILSALNWLTKDNQPGDVFFFLYSGHGSQQKDYTYLEDDGYNETILPCDHKTEGQIIDD
Colombia|30102100438-A     KNILSALNWLTKDNQPGDVFFFLYSGHGSQQKDYTYLEDDGYNETILPCDHKTEGQIIDD
Colombia|30101099036       KNILSALNWLTKDNQPGDVFFFLYSGHGSQQKDYTYLEDDGYNETILPCDHKTEGQIIDD
Colombia|30101099040       KNILSALNWLTKDNQPGDVFFFLYSGHGSQQKDYTYLEDDGYNETILPCDHKTEGQIIDD
Colombia|30102100486       KNILSALNWLTKDNQPGDVFFFLYSGHGSQQKDYTYLEDDGYNETILPCDHKTEGQIIDD
Colombia|30111110026       KNILSALNWLTKDNQPGDVFFFLYSGHGSQQKDYTYLEDDGYNETILPCDHKTEGQIIDD
Colombia|30103103280       KNILSALNWLTKDNQPGDVFFFLYSGHGSQQKDYTYLEDDGYNETILPCDHKTEGQIIDD
Mexico|330-A               KNILSALNWLTKDNQPGDVFFFLYSGHGSQQKDYTYLEDDGYNETILPCDHKTEGQIIDD
Mexico|938-A               KNILSALNWLTKDNQPGDVFFFLYSGHGSQQKDYTYLEDDGYNETILPCDHKTEGQIIDD
Mexico|Mexico_161-04       KNILSALNWLTKDNQPGDVFFFLYSGHGSQQKDYTYLEDDGYNETILPCDHKTEGQIIDD
Mexico|55-03               KNILSALNWLTKDNQPGDVFFFLYSGHGSQQKDYTYLEDDGYNETILPCDHKTEGQIIDD
Mexico|1086-A              KNILSALNWLTKDNQPGDVFFFLYSGHGSQQKDYTYLEDDGYNETILPCDHKTEGQIIDD
Mexico|203-04              KNILSALNWLTKDNQPGDVFFFLYSGHGSQQKDYTYLEDDGYNETILPCDHKTEGQIIDD
Mexico|63-08               KNILSALNWLTKDNQPGDVFFFLYSGHGSQQKDYTYLEDDGYNETILPCDHKTEGQIIDD
Mexico|21-A                KNILSALNWLTKDNQPGDVFFFLYSGHGSQQKDYTYLEDDGYNETILPCDHKTEGQIIDD
Mexico|566-A               KNILSALNWLTKDNQPGDVFFFLYSGHGSQQKDYTYLEDDGYNETILPCDHKTEGQIIDD
Mexico|32-E-03             KNILSALNWLTKDNQPGDVFFFLYSGHGSQQKDYTYLEDDGYNETILPCDHKTEGQIIDD
Mexico|760-A               KNILSALNWLTKDNQPGDVFFFLYSGHGSQQKDYTYLEDDGYNETILPCDHKTEGQIIDD
Mexico|118-A               KNILSALNWLTKDNQPGDVFFFLYSGHGSQQKDYTYLEDDGYNETILPCDHKTEGQIIDD
Mexico|165-A               KNILSALNWLTKDNQPGDVFFFLYSGHGSQQKDYTYLEDDGYNETILPCDHKTEGQIIDD
Mexico|267-A               KNILSALNWLTKDNQPGDVFFFLYSGHGSQQKDYTYLEDDGYNETILPCDHKTEGQIIDD
Peru|260                   KNILSALNWLTKDNQPGDVFFFLYSGHGSQQKDYTYLEDDGYNETILPCDHKTEGQIIDD
Peru|858                   KNILSALNWLTKDNQPGDVFFFLYSGHGSQQKDYTYLEDDGYNETILPCDHKTEGQIIDD
Peru|00692                 KNILSALNWLTKDNQPGDVFFFLYSGHGSQQKDYTYLEDDGYNETILPCDHKTEGQIIDD
Peru|07                    KNILSALNWLTKDNQPGDVFFFLYSGHGSQQKDYTYLEDDGYNETILPCDHKTEGQIIDD
Peru|00699                 KNILSALNWLTKDNQPGDVFFFLYSGHGSQQKDYTYLEDDGYNETILPCDHKTEGQIIDD
Peru|DTS0839               KNILSALNWLTKDNQPGDVFFFLYSGHGSQQKDYTYLEDDGYNETILPCDHKTEGQIIDD
```

Peru|00622	KNILSALNWLTKDNQPGDVFFFLYSGHGSQQKDYTYLEDDGYNETILPCDHKTEGQIIDD
Peru|99622	KNILSALNWLTKDNQPGDVFFFLYSGHGSQQKDYTYLEDDGYNETILPCDHKTEGQIIDD
Peru|ST02	KNILSALNWLTKDNQPGDVFFFLYSGHGSQQKDYTYLEDDGYNETILPCDHKTEGQIIDD
Peru|08	KNILSALNWLTKDNQPGDVFFFLYSGHGSQQKDYTYLEDDGYNETILPCDHKTEGQIIDD
Peru|06	KNILSALNWLTKDNQPGDVFFFLYSGHGSQQKDYTYLEDDGYNETILPCDHKTEGQIIDD
Peru|3136	KNILSALNWLTKDNQPGDVFFFLYSGHGSQQKDYTYLEDDGYNETILPCDHKTEGQIIDD
Peru|DTS0830	KNILSALNWLTKDNQPGDVFFFLYSGHGSQQKDYTYLEDDGYNETILPCDHKTEGQIIDD
India|PvHMP3	KNILSALNWLTKDNQPGDVFFFLYSGHGSQQKDYTYLEDDGYNETILPCDHKTEGQIIDD
Thailand|PvT01	KNILSALNWLTKDNQPGDVFFFLYSGHGSQQKDYTYLEDDGYNETILPCDHKTEGQIIDD
Papua|PvP01	KNILSALNWLTKDNQPGDVFFFLYSGHGSQQKDYTYLEDDGYNETILPCDHKTEGQIIDD
Brazil|BrazilI	KNILSALNWLTKDNQPGDVFFFLYSGHGSQQKDYTYLEDDGYNETILPCDHKTEGQIIDD
Brazil|Brazil29	KNILSALNWLTKDNQPGDVFFFLYSGHGSQQKDYTYLEDDGYNETILPCDHKTEGQIIDD
Brazil|Brazil32	KNILSALNWLTKDNQPGDVFFFLYSGHGSQQKDYTYLEDDGYNETILPCDHKTEGQIIDD
Colombia|30102100441-A	KNILSALNWLTKDNQPGDVFFFLYSGHGSQQKDYTYLEDDGYNETILPCDHKTEGQIIDD
Colombia|30111110015	KNILSALNWLTKDNQPGDVFFFLYSGHGSQQKDYTYLEDDGYNETILPCDHKTEGQIIDD
Colombia|30102100448	KNILSALNWLTKDNQPGDVFFFLYSGHGSQQKDYTYLEDDGYNETILPCDHKTEGQIIDD
Colombia|30102100485	KNILSALNWLTKDNQPGDVFFFLYSGHGSQQKDYTYLEDDGYNETILPCDHKTEGQIIDD
Colombia|30102100446	KNILSALNWLTKDNQPGDVFFFLYSGHGSQQKDYTYLEDDGYNETILPCDHKTEGQIIDD
Colombia|30111110020	KNILSALNWLTKDNQPGDVFFFLYSGHGSQQKDYTYLEDDGYNETILPCDHKTEGQIIDD
Colombia|30102100441-B	KNILSALNWLTKDNQPGDVFFFLYSGHGSQQKDYTYLEDDGYNETILPCDHKTEGQIIDD
Colombia|30102100439	KNILSALNWLTKDNQPGDVFFFLYSGHGSQQKDYTYLEDDGYNETILPCDHKTEGQIIDD
Colombia|30102100445	KNILSALNWLTKDNQPGDVFFFLYSGHGSQQKDYTYLEDDGYNETILPCDHKTEGQIIDD
Colombia|30102100440	KNILSALNWLTKDNQPGDVFFFLYSGHGSQQKDYTYLEDDGYNETILPCDHKTEGQIIDD
Colombia|30102100489	KNILSALNWLTKDNQPGDVFFFLYSGHGSQQKDYTYLEDDGYNETILPCDHKTEGQIIDD
Colombia|30102100488	KNILSALNWLTKDNQPGDVFFFLYSGHGSQQKDYTYLEDDGYNETILPCDHKTEGQIIDD
Colombia|30102100504	KNILSALNWLTKDNQPGDVFFFLYSGHGSQQKDYTYLEDDGYNETILPCDHKTEGQIIDD
Mexico|980-A	KNILSALNWLTKDNQPGDVFFFLYSGHGSQQKDYTYLEDDGYNETILPCDHKTEGQIIDD
Peru|3133	KNILSALNWLTKDNQPGDVFFFLYSGHGSQQKDYTYLEDDGYNETILPCDHKTEGQIIDD
Peru|259	KNILSALNWLTKDNQPGDVFFFLYSGHGSQQKDYTYLEDDGYNETILPCDHKTEGQIIDD
Peru|DTS0721	KNILSALNWLTKDNQPGDVFFFLYSGHGSQQKDYTYLEDDGYNETILPCDHKTEGQIIDD
Peru|1008	KNILSALNWLTKDNQPGDVFFFLYSGHGSQQKDYTYLEDDGYNETILPCDHKTEGQIIDD
Peru|3232	KNILSALNWLTKDNQPGDVFFFLYSGHGSQQKDYTYLEDDGYNETILPCDHKTEGQIIDD
Peru|DTS0791	KNILSALNWLTKDNQPGDVFFFLYSGHGSQQKDYTYLEDDGYNETILPCDHKTEGQIIDD
Peru|257	KNILSALNWLTKDNQPGDVFFFLYSGHGSQQKDYTYLEDDGYNETILPCDHKTEGQIIDD
Peru|2025	KNILSALNWLTKDNQPGDVFFFLYSGHGSQQKDYTYLEDDGYNETILPCDHKTEGQIIDD
Peru|4023	KNILSALNWLTKDNQPGDVFFFLYSGHGSQQKDYTYLEDDGYNETILPCDHKTEGQIIDD
Madagascar|M15	KNILSALNWLTKDNQPGDVFFFLYSGHGSQQKDYTYLEDDGYNETILPCDHKTEGQIIDD
Madagascar|M19	KNILSALNWLTKDNQPGDVFFFLYSGHGSQQKDYTYLEDDGYNETILPCDHKTEGQIIDD
Madagascar|M08	KNILSALNWLTKDNQPGDVFFFLYSGHGSQQKDYTYLEDDGYNETILPCDHKTEGQIIDD
Mauritania|MauritaniaI	KNILSALNWLTKDNQPGDVFFFLYSGHGSQQKDYTYLEDDGYNETILPCDHKTEGQIIDD
Cambodia|C127	KNILSALNWLTKDNQPGDVFFFLYSGHGSQQKDYTYLEDDGYNETILPCDHKTEGQIIDD
Cambodia|C08	KNILSALNWLTKDNQPGDVFFFLYSGHGSQQKDYTYLEDDGYNETILPCDHKTEGQIIDD
Cambodia|C15	KNILSALNWLTKDNQPGDVFFFLYSGHGSQQKDYTYLEDDGYNETILPCDHKTEGQIIDD
China|PvC01	KNILSALNWLTKDNQPGDVFFFLYSGHGSQQKDYTYLEDDGYNETILPCDHKTEGQIIDD
China|CMB1	KNILSALNWLTKDNQPGDVFFFLYSGHGSQQKDYTYLEDDGYNETILPCDHKTEGQIIDD
China|LZCH-20	KNILSALNWLTKDNQPGDVFFFLYSGHGSQQKDYTYLEDDGYNETILPCDHKTEGQIIDD
China|NB-16	KNILSALNWLTKDNQPGDVFFFLYSGHGSQQKDYTYLEDDGYNETILPCDHKTEGQIIDD
China|NB-15	KNILSALNWLTKDNQPGDVFFFLYSGHGSQQKDYTYLEDDGYNETILPCDHKTEGQIIDD
China|NB-13-1	KNILSALNWLTKDNQPGDVFFFLYSGHGSQQKDYTYLEDDGYNETILPCDHKTEGQIIDD
China|NB-17	KNILSALNWLTKDNQPGDVFFFLYSGHGSQQKDYTYLEDDGYNETILPCDHKTEGQIIDD
China|LZCH-4	KNILSALNWLTKDNQPGDVFFFLYSGHGSQQKDYTYLEDDGYNETILPCDHKTEGQIIDD
China|LZCH-13	KNILSALNWLTKDNQPGDVFFFLYSGHGSQQKDYTYLEDDGYNETILPCDHKTEGQIIDD
India|VII	KNILSALNWLTKDNQPGDVFFFLYSGHGSQQKDYTYLEDDGYNETILPCDHKTEGQIIDD
India|NYC	KNILSALNWLTKDNQPGDVFFFLYSGHGSQQKDYTYLEDDGYNETILPCDHKTEGQIIDD
North|NorthKorean	KNILSALNWLTKDNQPGDVFFFLYSGHGSQQKDYTYLEDDGYNETILPCDHKTEGQIIDD
Thailand|VKBT-106	KNILSALNWLTKDNQPGDVFFFLYSGHGSQQKDYTYLEDDGYNETILPCDHKTEGQIIDD
Thailand|VKBT-98	KNILSALNWLTKDNQPGDVFFFLYSGHGSQQKDYTYLEDDGYNETILPCDHKTEGQIIDD
Thailand|VKTS-39	KNILSALNWLTKDNQPGDVFFFLYSGHGSQQKDYTYLEDDGYNETILPCDHKTEGQIIDD
Thailand|VKTS-36	KNILSALNWLTKDNQPGDVFFFLYSGHGSQQKDYTYLEDDGYNETILPCDHKTEGQIIDD
Thailand|VKTS-52	KNILSALNWLTKDNQPGDVFFFLYSGHGSQQKDYTYLEDDGYNETILPCDHKTEGQIIDD
Thailand|VKBT-100	KNILSALNWLTKDNQPGDVFFFLYSGHGSQQKDYTYLEDDGYNETILPCDHKTEGQIIDD
Thailand|VKTS-45	KNILSALNWLTKDNQPGDVFFFLYSGHGSQQKDYTYLEDDGYNETILPCDHKTEGQIIDD
Thailand|VKBT-95	KNILSALNWLTKDNQPGDVFFFLYSGHGSQQKDYTYLEDDGYNETILPCDHKTEGQIIDD
Thailand|VKBT-72	KNILSALNWLTKDNQPGDVFFFLYSGHGSQQKDYTYLEDDGYNETILPCDHKTEGQIIDD
Thailand|VKBT-94	KNILSALNWLTKDNQPGDVFFFLYSGHGSQQKDYTYLEDDGYNETILPCDHKTEGQIIDD
Thailand|VKBT-99	KNILSALNWLTKDNQPGDVFFFLYSGHGSQQKDYTYLEDDGYNETILPCDHKTEGQIIDD
Thailand|VKBT-101	KNILSALNWLTKDNQPGDVFFFLYSGHGSQQKDYTYLEDDGYNETILPCDHKTEGQIIDD
Thailand|VKBT-71	KNILSALNWLTKDNQPGDVFFFLYSGHGSQQKDYTYLEDDGYNETILPCDHKTEGQIIDD
Thailand|VKTS-37	KNILSALNWLTKDNQPGDVFFFLYSGHGSQQKDYTYLEDDGYNETILPCDHKTEGQIIDD
Papua|Chesson	KNILSALNWLTKDNQPGDVFFFLYSGHGSQQKDYTYLEDDGYNETILPCDHKTEGQIIDD
Papua|PNG58	KNILSALNWLTKDNQPGDVFFFLYSGHGSQQKDYTYLEDDGYNETILPCDHKTEGQIIDD
Papua|PVRVL1997	KNILSALNWLTKDNQPGDVFFFLYSGHGSQQKDYTYLEDDGYNETILPCDHKTEGQIIDD
Papua|PNG72	KNILSALNWLTKDNQPGDVFFFLYSGHGSQQKDYTYLEDDGYNETILPCDHKTEGQIIDD
Papua|XUC014	KNILSALNWLTKDNQPGDVFFFLYSGHGSQQKDYTYLEDDGYNETILPCDHKTEGQIIDD

```
Gabon|Pvl10                 KNILSALNWLTKDNQPGDVFFFLYSGHGSQQKDYTYLEDDGYNETILPCDHKTEGQIIDD
Cameroon|PvSY56             KNILSALNWLTKDNEPGDVFFFLYSGHGSQQKDYTYLEDDGYNETILPCDHKTEGQIIDD
Cameroon|PvSY42             KNILSALNWLTKDNEPGDVFFFLYSGHGSQQKDYTYLEDDGYNETILPCDHKTEGQIIDD
Cameroon|PvSY43             KNILSALNWLTKDNEPGDVFFFLYSGHGSQQKDYTYLEDDGYNETILPCDHKTEGQIIDD
Gabon|Pvl06                 KNILSALNWLTKDNEPGDVFFFLYSGHGSQQKDYTYLEDDGYNETILPCDHKTEGQIIDD
Gabon|Pvl11                 KNILSALNWLTKDNEPGDVFFFLYSGHGSQQKDYTYLEDDGYNETILPCDHKTEGQIIDD
Gabon|Pvl03                 KNILSALNWLTKDNEPGDVFFFLYSGHGSQQKDYTYLEDDGYNETILPCDHKTEGQIIDD
Gabon|Pvl04                 KNILSALNWLTKDNEPGDVFFFLYSGHGSQQKDYTYLEDDGYNETILPCDHKTEGQIIDD
                            **************:*********************************************

                           406                                                         465
ElSalvador|Sal1             ELHRFLVQPLNDGVKLIAVMDCCNAGSCIDLAYKYLKSKKWKEVKNPFHVVCDVSQFSG
Colombia|30102100437        ELHRFLVQPLNDGVKLIAVMDCCNAGSCIDLAYKYLKSKKWKEVKNPFHVVCDVSQFSG
Colombia|30102100490        ELHRFLVQPLNDGVKLIAVMDCCNAGSCIDLAYKYLKSKKWKEVKNPFHVVCDVSQFSG
Colombia|30102100438-B      ELHRFLVQPLNDGVKLIAVMDCCNAGSCIDLAYKYLKSKKWKEVKNPFHVVCDVSQFSG
Colombia|30102100438-A      ELHRFLVQPLNDGVKLIAVMDCCNAGSCIDLAYKYLKSKKWKEVKNPFHVVCDVSQFSG
Colombia|30101099036        ELHRFLVQPLNDGVKLIAVMDCCNAGSCIDLAYKYLKSKKWKEVKNPFHVVCDVSQFSG
Colombia|30101099040        ELHRFLVQPLNDGVKLIAVMDCCNAGSCIDLAYKYLKSKKWKEVKNPFHVVCDVSQFSG
Colombia|30102100486        ELHRFLVQPLNDGVKLIAVMDCCNAGSCIDLAYKYLKSKKWKEVKNPFHVVCDVSQFSG
Colombia|30111110026        ELHRFLVQPLNDGVKLIAVMDCCNAGSCIDLAYKYLKSKKWKEVKNPFHVVCDVSQFSG
Colombia|30103103280        ELHRFLVQPLNDGVKLIAVMDCCNAGSCIDLAYKYLKSKKWKEVKNPFHVVCDVSQFSG
Mexico|330-A                ELHRFLVQPLNDGVKLIAVMDCCNAGSCIDLAYKYLKSKKWKEVKNPFHVVCDVSQFSG
Mexico|938-A                ELHRFLVQPLNDGVKLIAVMDCCNAGSCIDLAYKYLKSKKWKEVKNPFHVVCDVSQFSG
Mexico|Mexico_161-04        ELHRFLVQPLNDGVKLIAVMDCCNAGSCIDLAYKYLKSKKWKEVKNPFHVVCDVSQFSG
Mexico|55-03                ELHRFLVQPLNDGVKLIAVMDCCNAGSCIDLAYKYLKSKKWKEVKNPFHVVCDVSQFSG
Mexico|1086-A               ELHRFLVQPLNDGVKLIAVMDCCNAGSCIDLAYKYLKSKKWKEVKNPFHVVCDVSQFSG
Mexico|203-04               ELHRFLVQPLNDGVKLIAVMDCCNAGSCIDLAYKYLKSKKWKEVKNPFHVVCDVSQFSG
Mexico|63-08                ELHRFLVQPLNDGVKLIAVMDCCNAGSCIDLAYKYLKSKKWKEVKNPFHVVCDVSQFSG
Mexico|21-A                 ELHRFLVQPLNDGVKLIAVMDCCNAGSCIDLAYKYLKSKKWKEVKNPFHVVCDVSQFSG
Mexico|566-A                ELHRFLVQPLNDGVKLIAVMDCCNAGSCIDLAYKYLKSKKWKEVKNPFHVVCDVSQFSG
Mexico|32-E-03              ELHRFLVQPLNDGVKLIAVMDCCNAGSCIDLAYKYLKSKKWKEVKNPFHVVCDVSQFSG
Mexico|760-A                ELHRFLVQPLNDGVKLIAVMDCCNAGSCIDLAYKYLKSKKWKEVKNPFHVVCDVSQFSG
Mexico|118-A                ELHRFLVQPLNDGVKLIAVMDCCNAGSCIDLAYKYLKSKKWKEVKNPFHVVCDVSQFSG
Mexico|165-A                ELHRFLVQPLNDGVKLIAVMDCCNAGSCIDLAYKYLKSKKWKEVKNPFHVVCDVSQFSG
Mexico|267-A                ELHRFLVQPLNDGVKLIAVMDCCNAGSCIDLAYKYLKSKKWKEVKNPFHVVCDVSQFSG
Peru|260                    ELHRFLVQPLNDGVKLIAVMDCCNAGSCIDLAYKYLKSKKWKEVKNPFHVVCDVSQFSG
Peru|858                    ELHRFLVQPLNDGVKLIAVMDCCNAGSCIDLAYKYLKSKKWKEVKNPFHVVCDVSQFSG
Peru|00692                  ELHRFLVQPLNDGVKLIAVMDCCNAGSCIDLAYKYLKSKKWKEVKNPFHVVCDVSQFSG
Peru|07                     ELHRFLVQPLNDGVKLIAVMDCCNAGSCIDLAYKYLKSKKWKEVKNPFHVVCDVSQFSG
Peru|00699                  ELHRFLVQPLNDGVKLIAVMDCCNAGSCIDLAYKYLKSKKWKEVKNPFHVVCDVSQFSG
Peru|DTS0839                ELHRFLVQPLNDGVKLIAVMDCCNAGSCIDLAYKYLKSKKWKEVKNPFHVVCDVSQFSG
Peru|00622                  ELHRFLVQPLNDGVKLIAVMDCCNAGSCIDLAYKYLKSKKWKEVKNPFHVVCDVSQFSG
Peru|99622                  ELHRFLVQPLNDGVKLIAVMDCCNAGSCIDLAYKYLKSKKWKEVKNPFHVVCDVSQFSG
Peru|ST02                   ELHRFLVQPLNDGVKLIAVMDCCNAGSCIDLAYKYLKSKKWKEVKNPFHVVCDVSQFSG
Peru|08                     ELHRFLVQPLNDGVKLIAVMDCCNAGSCIDLAYKYLKSKKWKEVKNPFHVVCDVSQFSG
Peru|06                     ELHRFLVQPLNDGVKLIAVMDCCNAGSCIDLAYKYLKSKKWKEVKNPFHVVCDVSQFSG
Peru|3136                   ELHRFLVQPLNDGVKLIAVMDCCNAGSCIDLAYKYLKSKKWKEVKNPFHVVCDVSQFSG
Peru|DTS0830                ELHRFLVQPLNDGVKLIAVMDCCNAGSCIDLAYKYLKSKKWKEVKNPFHVVCDVSQFSG
India|PvHMP3                ELHRFLVQPLNDGVKLIAVMDCCNAGSCIDLAYKYLKSKKWKEVKNPFHVVCDVSQFSG
Thailand|PvT01              ELHRFLVQPLNDGVKLIAVMDCCNAGSCIDLAYKYLKSKKWKEVKNPFHVVCDVSQFSG
Papua|PvP01                 ELHRFLVQPLNDGVKLIAVMDCCNAGSCIDLAYKYLKSKKWKEVKNPFHVVCDVSQFSG
Brazil|BrazilI              ELHRFLVQPLNDGVKLIAVMDCCNAGSCIDLAYKYLKSKKWKEVKNPFHVVCDVSQFSG
Brazil|Brazil29             ELHRFLVQPLNDGVKLIAVMDCCNAGSCIDLAYKYLKSKKWKEVKNPFHVVCDVSQFSG
Brazil|Brazil32             ELHRFLVQPLNDGVKLIAVMDCCNAGSCIDLAYKYLKSKKWKEVKNPFHVVCDVSQFSG
Colombia|30102100441-A      ELHRFLVQPLNDGVKLIAVMDCCNAGSCIDLAYKYLKSKKWKEVKNPFHVVCDVSQFSG
Colombia|30111110015        ELHRFLVQPLNDGVKLIAVMDCCNAGSCIDLAYKYLKSKKWKEVKNPFHVVCDVSQFSG
Colombia|30102100448        ELHRFLVQPLNDGVKLIAVMDCCNAGSCIDLAYKYLKSKKWKEVKNPFHVVCDVSQFSG
Colombia|30102100485        ELHRFLVQPLNDGVKLIAVMDCCNAGSCIDLAYKYLKSKKWKEVKNPFHVVCDVSQFSG
Colombia|30102100446        ELHRFLVQPLNDGVKLIAVMDCCNAGSCIDLAYKYLKSKKWKEVKNPFHVVCDVSQFSG
Colombia|30111110020        ELHRFLVQPLNDGVKLIAVMDCCNAGSCIDLAYKYLKSKKWKEVKNPFHVVCDVSQFSG
Colombia|30102100441-B      ELHRFLVQPLNDGVKLIAVMDCCNAGSCIDLAYKYLKSKKWKEVKNPFHVVCDVSQFSG
Colombia|30102100439        ELHRFLVQPLNDGVKLIAVMDCCNAGSCIDLAYKYLKSKKWKEVKNPFHVVCDVSQFSG
Colombia|30102100445        ELHRFLVQPLNDGVKLIAVMDCCNAGSCIDLAYKYLKSKKWKEVKNPFHVVCDVSQFSG
Colombia|30102100440        ELHRFLVQPLNDGVKLIAVMDCCNAGSCIDLAYKYLKSKKWKEVKNPFHVVCDVSQFSG
Colombia|30102100489        ELHRFLVQPLNDGVKLIAVMDCCNAGSCIDLAYKYLKSKKWKEVKNPFHVVCDVSQFSG
Colombia|30102100488        ELHRFLVQPLNDGVKLIAVMDCCNAGSCIDLAYKYLKSKKWKEVKNPFHVVCDVSQFSG
Colombia|30102100504        ELHRFLVQPLNDGVKLIAVMDCCNAGSCIDLAYKYLKSKKWKEVKNPFHVVCDVSQFSG
Mexico|980-A                ELHRFLVQPLNDGVKLIAVMDCCNAGSCIDLAYKYLKSKKWKEVKNPFHVVCDVSQFSG
Peru|3133                   ELHRFLVQPLNDGVKLIAVMDCCNAGSCIDLAYKYLKSKKWKEVKNPFHVVCDVSQFSG
Peru|259                    ELHRFLVQPLNDGVKLIAVMDCCNAGSCIDLAYKYLKSKKWKEVKNPFHVVCDVSQFSG
Peru|DTS0721                ELHRFLVQPLNDGVKLIAVMDCCNAGSCIDLAYKYLKSKKWKEVKNPFHVVCDVSQFSG
Peru|1008                   ELHRFLVQPLNDGVKLIAVMDCCNAGSCIDLAYKYLKSKKWKEVKNPFHVVCDVSQFSG
Peru|3232                   ELHRFLVQPLNDGVKLIAVMDCCNAGSCIDLAYKYLKSKKWKEVKNPFHVVCDVSQFSG
Peru|DTS0791                ELHRFLVQPLNDGVKLIAVMDCCNAGSCIDLAYKYLKSKKWKEVKNPFHVVCDVSQFSG
Peru|257                    ELHRFLVQPLNDGVKLIAVMDCCNAGSCIDLAYKYLKSKKWKEVKNPFHVVCDVSQFSG
```

```
Peru|2025                   ELHRFLVQPLNDGVKLIAVMDCCNAGSCIDLAYKYLKLSKKWKEVKNPFHVVCDVSQFSG
Peru|4023                   ELHRFLVQPLNDGVKLIAVMDCCNAGSCIDLAYKYLKLSKKWKEVKNPFHVVCDVSQFSG
Madagascar|M15              ELHRFLVQPLNDGVKLIAVMDCCNAGSCIDLAYKYLKLSKKWKEVKNPFHVVCDVSQFSG
Madagascar|M19              ELHRFLVQPLNDGVKLIAVMDCCNAGSCIDLAYKYLKLSKKWKEVKNPFHVVCDVSQFSG
Madagascar|M08              ELHRFLVQPLNDGVKLIAVMDCCNAGSCIDLAYKYLKLSKKWKEVKNPFHVVCDVSQFSG
Mauritania|MauritaniaI      ELHRFLVQPLNDGVKLIAVMDCCNAGSCIDLAYKYLKLSKKWKEVKNPFHVVCDVSQFSG
Cambodia|C127               ELHRFLVQPLNDGVKLIAVMDCCNAGSCIDLAYKYLKLSKKWKEVKNPFHVVCDVSQFSG
Cambodia|C08                ELHRFLVQPLNDGVKLIAVMDCCNAGSCIDLAYKYLKLSKKWKEVKNPFHVVCDVSQFSG
Cambodia|C15                ELHRFLVQPLNDGVKLIAVMDCCNAGSCIDLAYKYLKLSKKWKEVKNPFHVVCDVSQFSG
China|PvC01                 ELHRFLVQPLNDGVKLIAVMDCCNAGSCIDLAYKYLKLSKKWKEVKNPFHVVCDVSQFSG
China|CMB1                  ELHRFLVQPLNDGVKLIAVMDCCNAGSCIDLAYKYLKLSKKWKEVKNPFHVVCDVSQFSG
China|LZCH-20               ELHRFLVQPLNDGVKLIAVMDCCNAGSCIDLAYKYLKLSKKWKEVKNPFHVVCDVSQFSG
China|NB-16                 ELHRFLVQPLNDGVKLIAVMDCCNAGSCIDLAYKYLKLSKKWKEVKNPFHVVCDVSQFSG
China|NB-15                 ELHRFLVQPLNDGVKLIAVMDCCNAGSCIDLAYKYLKLSKKWKEVKNPFHVVCDVSQFSG
China|NB-13-1               ELHRFLVQPLNDGVKLIAVMDCCNAGSCIDLAYKYLKLSKKWKEVKNPFHVVCDVSQFSG
China|NB-17                 ELHRFLVQPLNDGVKLIAVMDCCNAGSCIDLAYKYLKLSKKWKEVKNPFHVVCDVSQFSG
China|LZCH-4                ELHRFLVQPLNDGVKLIAVMDCCNAGSCIDLAYKYLKLSKKWKEVKNPFHVVCDVSQFSG
China|LZCH-13               ELHRFLVQPLNDGVKLIAVMDCCNAGSCIDLAYKYLKLSKKWKEVKNPFHVVCDVSQFSG
India|VII                   ELHRFLVQPLNDGVKLIAVMDCCNAGSCIDLAYKYLKLSKKWKEVKNPFHVVCDVSQFSG
India|NYC                   ELHRFLVQPLNDGVKLIAVMDCCNAGSCIDLAYKYLKLSKKWKEVKNPFHVVCDVSQFSG
North|NorthKorean           ELHRFLVQPLNDGVKLIAVMDCCNAGSCIDLAYKYLKLSKKWKEVKNPFHVVCDVSQFSG
Thailand|VKBT-106           ELHRFLVQPLNDGVKLIAVMDCCNAGSCIDLAYKYLKLSKKWKEVKNPFHVVCDVSQFSG
Thailand|VKBT-98            ELHRFLVQPLNDGVKLIAVMDCCNAGSCIDLAYKYLKLSKKWKEVKNPFHVVCDVSQFSG
Thailand|VKTS-39            ELHRFLVQPLNDGVKLIAVMDCCNAGSCIDLAYKYLKLSKKWKEVKNPFHVVCDVSQFSG
Thailand|VKTS-36            ELHRFLVQPLNDGVKLIAVMDCCNAGSCIDLAYKYLKLSKKWKEVKNPFHVVCDVSQFSG
Thailand|VKTS-52            ELHRFLVQPLNDGVKLIAVMDCCNAGSCIDLAYKYLKLSKKWKEVKNPFHVVCDVSQFSG
Thailand|VKBT-100           ELHRFLVQPLNDGVKLIAVMDCCNAGSCIDLAYKYLKLSKKWKEVKNPFHVVCDVSQFSG
Thailand|VKTS-45            ELHRFLVQPLNDGVKLIAVMDCCNAGSCIDLAYKYLKLSKKWKEVKNPFHVVCDVSQFSG
Thailand|VKBT-95            ELHRFLVQPLNDGVKLIAVMDCCNAGSCIDLAYKYLKLSKKWKEVKNPFHVVCDVSQFSG
Thailand|VKBT-72            ELHRFLVQPLNDGVKLIAVMDCCNAGSCIDLAYKYLKLSKKWKEVKNPFHVVCDVSQFSG
Thailand|VKBT-94            ELHRFLVQPLNDGVKLIAVMDCCNAGSCIDLAYKYLKLSKKWKEVKNPFHVVCDVSQFSG
Thailand|VKBT-99            ELHRFLVQPLNDGVKLIAVMDCCNAGSCIDLAYKYLKLSKKWKEVKNPFHVVCDVSQFSG
Thailand|VKBT-101           ELHRFLVQPLNDGVKLIAVMDCCNAGSCIDLAYKYLKLSKKWKEVKNPFHVVCDVSQFSG
Thailand|VKBT-71            ELHRFLVQPLNDGVKLIAVMDCCNAGSCIDLAYKYLKLSKKWKEVKNPFHVVCDVSQFSG
Thailand|VKTS-37            ELHRFLVQPLNDGVKLIAVMDCCNAGSCIDLAYKYLKLSKKWKEVKNPFHVVCDVSQFSG
Papua|Chesson               ELHRFLVQPLNDGVKLIAVMDCCNAGSCIDLAYKYLKLSKKWKEVKNPFHVVCDVSQFSG
Papua|PNG58                 ELHRFLVQPLNDGVKLIAVMDCCNAGSCIDLAYKYLKLSKKWKEVKNPFHVVCDVSQFSG
Papua|PVRVL1997             ELHRFLVQPLNDGVKLIAVMDCCNAGSCIDLAYKYLKLSKKWKEVKNPFHVVCDVSQFSG
Papua|PNG72                 ELHRFLVQPLNDGVKLIAVMDCCNAGSCIDLAYKYLKLSKKWKEVKNPFHVVCDVSQFSG
Papua|XUC014                ELHRFLVQPLNDGVKLIAVMDCCNAGSCIDLAYKYLKLSKKWKEVKNPFHVVCDVSQFSG
Gabon|Pvl10                 ELHRFLVQPLNDGVKLIAVMDCCNAGSCIDLAYKYLKLSKKWKEVKNPFHVVCDVSQFSG
Cameroon|PvSY56             ELHRFLVQPLNDGVKLIAVMDCCNAGSCIDLAYKYLKLSKKWKEVKNPFHVVCDVSQFSG
Cameroon|PvSY42             ELHRFLVQPLNDGVKLIAVMDCCNAGSCIDLAYKYLKLSKKWKEVKNPFHVVCDVSQFSG
Cameroon|PvSY43             ELHRFLVQPLNDGVKLIAVMDCCNAGSCIDLAYKYLKLSKKWKEVKNPFHVVCDVSQFSG
Gabon|Pvl06                 ELHRFLVQPLNDGVKLIAVMDCCNAGSCIDLAYKYLKLSKKWKEVKNPFHVVCDVSQFSG
Gabon|Pvl11                 ELHRFLVQPLNDGVKLIAVMDCCNAGSCIDLAYKYLKLSKKWKEVKNPFHVVCDVSQFSG
Gabon|Pvl03                 ELHRFLVQPLNDGVKLIAVMDCCNAGSCIDLAYKYLKLSKKWKEVKNPFHVVCDVSQFSG
Gabon|Pvl04                 ELHRFLVQPLNDGVKLIAVMDCCNAGSCIDLAYKYLKLSKKWKEVKNPFHVMCDVSQFSG
                            ***************************************************:********

                            466                            497
ElSalvador|Sal1             CKDMEFSREIDTGKHAPGGALVTAMIHVLGAS
Colombia|30102100437        CKDMEFSREIDTGKHAPGGALVTAMIHVLGAS
Colombia|30102100490        CKDMEFSREIDTGKHAPGGALVTAMIHVLGAS
Colombia|30102100438-B      CKDMEFSREIDTGKHAPGGALVTAMIHVLGAS
Colombia|30102100438-A      CKDMEFSREIDTGKHAPGGALVTAMIHVLGAS
Colombia|30101099036        CKDMEFSREIDTGKHAPGGALVTAMIHVLGAS
Colombia|30101099040        CKDMEFSREIDTGKHAPGGALVTAMIHVLGAS
Colombia|30102100486        CKDMEFSREIDTGKHAPGGALVTAMIHVLGAS
Colombia|30111110026        CKDMEFSREIDTGKHAPGGALVTAMIHVLGAS
Colombia|30103103280        CKDMEFSREIDTGKHAPGGALVTAMIHVLGAS
Mexico|330-A                CKDMEFSREIDTGKHAPGGALVTAMIHVLGAS
Mexico|938-A                CKDMEFSREIDTGKHAPGGALVTAMIHVLGAS
Mexico|Mexico_161-04        CKDMEFSREIDTGKHAPGGALVTAMIHVLGAS
Mexico|55-03                CKDMEFSREIDTGKHAPGGALVTAMIHVLGAS
Mexico|1086-A               CKDMEFSREIDTGKHAPGGALVTAMIHVLGAS
Mexico|203-04               CKDMEFSREIDTGKHAPGGALVTAMIHVLGAS
Mexico|63-08                CKDMEFSREIDTGKHAPGGALVTAMIHVLGAS
Mexico|21-A                 CKDMEFSREIDTGKHAPGGALVTAMIHVLGAS
Mexico|566-A                CKDMEFSREIDTGKHAPGGALVTAMIHVLGAS
Mexico|32-E-03              CKDMEFSREIDTGKHAPGGALVTAMIHVLGAS
Mexico|760-A                CKDMEFSREIDTGKHAPGGALVTAMIHVLGAS
Mexico|118-A                CKDMEFSREIDTGKHAPGGALVTAMIHVLGAS
Mexico|165-A                CKDMEFSREIDTGKHAPGGALVTAMIHVLGAS
Mexico|267-A                CKDMEFSREIDTGKHAPGGALVTAMIHVLGAS
```

Peru|260	CKDMEFSREIDTGKHAPGGALVTAMIHVLGAS
Peru|858	CKDMEFSREIDTGKHAPGGALVTAMIHVLGAS
Peru|00692	CKDMEFSREIDTGKHAPGGALVTAMIHVLGAS
Peru|07	CKDMEFSREIDTGKHAPGGALVTAMIHVLGAS
Peru|00699	CKDMEFSREIDTGKHAPGGALVTAMIHVLGAS
Peru|DTS0839	CKDMEFSREIDTGKHAPGGALVTAMIHVLGAS
Peru|00622	CKDMEFSREIDTGKHAPGGALVTAMIHVLGAS
Peru|99622	CKDMEFSREIDTGKHAPGGALVTAMIHVLGAS
Peru|ST02	CKDMEFSREIDTGKHAPGGALVTAMIHVLGAS
Peru|08	CKDMEFSREIDTGKHAPGGALVTAMIHVLGAS
Peru|06	CKDMEFSREIDTGKHAPGGALVTAMIHVLGAS
Peru|3136	CKDMEFSREIDTGKHAPGGALVTAMIHVLGAS
Peru|DTS0830	CKDMEFSREIDTGKHAPGGALVTAMIHVLGAS
India|PvHMP3	CKDMEFSREIDTGKHAPGGALVTAMIHVLGAS
Thailand|PvT01	CKDMEFSREIDTGKHAPGGALVTAMIHVLGAS
Papua|PvP01	CKDMEFSREIDTGKHAPGGALVTAMIHVLGAS
Brazil|BrazilI	CKDMEFSREIDTGRHAPGGALVTAMIHVLGAS
Brazil|Brazil29	CKDMEFSREIDTGRHAPGGALVTAMIHVLGAS
Brazil|Brazil32	CKDMEFSREIDTGRHAPGGALVTAMIHVLGAS
Colombia|30102100441-A	CKDMEFSREIDTGRHAPGGALVTAMIHVLGAS
Colombia|30111110015	CKDMEFSREIDTGRHAPGGALVTAMIHVLGAS
Colombia|30102100448	CKDMEFSREIDTGRHAPGGALVTAMIHVLGAS
Colombia|30102100485	CKDMEFSREIDTGRHAPGGALVTAMIHVLGAS
Colombia|30102100446	CKDMEFSREIDTGRHAPGGALVTAMIHVLGAS
Colombia|30111110020	CKDMEFSREIDTGRHAPGGALVTAMIHVLGAS
Colombia|30102100441-B	CKDMEFSREIDTGRHAPGGALVTAMIHVLGAS
Colombia|30102100439	CKDMEFSREIDTGRHAPGGALVTAMIHVLGAS
Colombia|30102100445	CKDMEFSREIDTGRHAPGGALVTAMIHVLGAS
Colombia|30102100440	CKDMEFSREIDTGRHAPGGALVTAMIHVLGAS
Colombia|30102100489	CKDMEFSREIDTGRHAPGGALVTAMIHVLGAS
Colombia|30102100488	CKDMEFSREIDTGRHAPGGALVTAMIHVLGAS
Colombia|30102100504	CKDMEFSREIDTGRHAPGGALVTAMIHVLGAS
Mexico|980-A	CKDMEFSREIDTGRHAPGGALVTAMIHVLGAS
Peru|3133	CKDMEFSREIDTGRHAPGGALVTAMIHVLGAS
Peru|259	CKDMEFSREIDTGRHAPGGALVTAMIHVLGAS
Peru|DTS0721	CKDMEFSREIDTGRHAPGGALVTAMIHVLGAS
Peru|1008	CKDMEFSREIDTGRHAPGGALVTAMIHVLGAS
Peru|3232	CKDMEFSREIDTGRHAPGGALVTAMIHVLGAS
Peru|DTS0791	CKDMEFSREIDTGRHAPGGALVTAMIHVLGAS
Peru|257	CKDMEFSREIDTGRHAPGGALVTAMIHVLGAS
Peru|2025	CKDMEFSREIDTGRHAPGGALVTAMIHVLGAS
Peru|4023	CKDMEFSREIDTGRHAPGGALVTAMIHVLGAS
Madagascar|M15	CKDMEFSREIDTGRHAPGGALVTAMIHVLGAS
Madagascar|M19	CKDMEFSREIDTGRHAPGGALVTAMIHVLGAS
Madagascar|M08	CKDMEFSREIDTGRHAPGGALVTAMIHVLGAS
Mauritania|MauritaniaI	CKDMEFSREIDTGRHAPGGALVTAMIHVLGAS
Cambodia|C127	CKDMEFSREIDTGRHAPGGALVTAMIHVLGAS
Cambodia|C08	CKDMEFSREIDTGRHAPGGALVTAMIHVLGAS
Cambodia|C15	CKDMEFSREIDTGRHAPGGALVTAMIHVLGAS
China|PvC01	CKDMEFSREIDTGRHAPGGALVTAMIHVLGAS
China|CMB1	CKDMEFSREIDTGRHAPGGALVTAMIHVLGAS
China|LZCH-20	CKDMEFSREIDTGRHAPGGALVTAMIHVLGAS
China|NB-16	CKDMEFSREIDTGRHAPGGALVTAMIHVLGAS
China|NB-15	CKDMEFSREIDTGRHAPGGALVTAMIHVLGAS
China|NB-13-1	CKDMEFSREIDTGRHAPGGALVTAMIHVLGAS
China|NB-17	CKDMEFSREIDTGRHAPGGALVTAMIHVLGAS
China|LZCH-4	CKDMEFSREIDTGRHAPGGALVTAMIHVLGAS
China|LZCH-13	CKDMEFSREIDTGRHAPGGALVTAMIHVLGAS
India|VII	CKDMEFSREIDTGRHAPGGALVTAMIHVLGAS
India|NYC	CKDMEFSREIDTGRHAPGGALVTAMIHVLGAS
North|NorthKorean	CKDMEFSREIDTGRHAPGGALVTAMIHVLGAS
Thailand|VKBT-106	CKDMEFSREIDTGRHAPGGALVTAMIHVLGAS
Thailand|VKBT-98	CKDMEFSREIDTGRHAPGGALVTAMIHVLGAS
Thailand|VKTS-39	CKDMEFSREIDTGRHAPGGALVTAMIHVLGAS
Thailand|VKTS-36	CKDMEFSREIDTGRHAPGGALVTAMIHVLGAS
Thailand|VKTS-52	CKDMEFSREIDTGRHAPGGALVTAMIHVLGAS
Thailand|VKBT-100	CKDMEFSREIDTGRHAPGGALVTAMIHVLGAS
Thailand|VKTS-45	CKDMEFSREIDTGRHAPGGALVTAMIHVLGAS
Thailand|VKBT-95	CKDMEFSREIDTGRHAPGGALVTAMIHVLGAS
Thailand|VKBT-72	CKDMEFSREIDTGRHAPGGALVTAMIHVLGAS
Thailand|VKBT-94	CKDMEFSREIDTGRHAPGGALVTAMIHVLGAS
Thailand|VKBT-99	CKDMEFSREIDTGRHAPGGALVTAMIHVLGAS
Thailand|VKBT-101	CKDMEFSREIDTGRHAPGGALVTAMIHVLGAS
Thailand|VKBT-71	CKDMEFSREIDTGRHAPGGALVTAMIHVLGAS

```
Thailand|VKTS-37          CKDMEFSREIDTGRHAPGGALVTAMIHVLGAS
Papua|Chesson             CKDMEFSREIDTGRHAPGGALVTAMIHVLGAS
Papua|PNG58               CKDMEFSREIDTGRHAPGGALVTAMIHVLGAS
Papua|PVRVL1997           CKDMEFSREIDTGRHAPGGALVTAMIHVLGAS
Papua|PNG72               CKDMEFSREIDTGRHAPGGALVTAMIHVLGAS
Papua|XUC014              CKDMEFSREIDTGRHAPGGALVTAMIHVLGAS
Gabon|Pvl10               CKDMEFSREIDTGRHAPGGALVTAMIHVLGAS
Cameroon|PvSY56           CKDMEFSREIDTGRHAPGGALVTAMIHVLGAS
Cameroon|PvSY42           CKDMEFSREIDTGRHAPGGALVTAMIHVLGAS
Cameroon|PvSY43           CKDMEFSREIDTGRHAPGGALVTAMIHVLGAS
Gabon|Pvl06               CKDMEFSREIDTGRHAPGGALVTAMIHVLGAS
Gabon|Pvl11               CKDMEFSREIDTGRHAPGGALVTAMIHVLGAS
Gabon|Pvl03               CKDMEFSREIDTGRHAPGGALVTAMIHVLGAS
Gabon|Pvl04               CKDMEFSREIDTGRHAPGGALVTAMIHVLGAS
                          *************:******************
```

Multiple alignment of *Pv*MCA1 peptidase domain of *Plasmodium vivax* isolates from fifteen malaria-endemic countries worldwide. Amino acid sequences of Peptidase_C14 domain of *Pv*MCA1 were deduced from 112 *P. vivax* nucleotide genomic sequences available in GenBank and PlasmoDB and, then, aligned to identify polymorphisms. Four amino acid substitutions are identified, as indicates in yellow (L310V), blue (Q360E), green (V457M) and purple (K479R), with the first three substitutions restricted to ape-infecting isolates from Cameroon and Gabon.
